# Level of antiretroviral therapy adherence and associated factors during COVID-19 pandemic era in public hospitals of Jigjiga City eastern Ethiopia: a cross-sectional study

**DOI:** 10.3389/fpubh.2024.1363903

**Published:** 2024-05-24

**Authors:** Samson Tesfay, Firayad Ayele, Birhane Fissahaye, Haftu Asmerom, Berhe Gebremichael

**Affiliations:** ^1^Jigjiga Regional Health Bureau, Jigjiga, Ethiopia; ^2^School of Medical Laboratory Sciences, College of Health and Medical Sciences, Haramaya University, Harar, Ethiopia; ^3^School of Nursing and Midwifery, College of Health and Medical Sciences, Aksum University, Tigray, Ethiopia; ^4^School of Public Health, College of Health and Medical Sciences, Haramaya University, Harar, Ethiopia

**Keywords:** antiretroviral therapy (ART), adherence, HIV, COVID-19, eastern Ethiopia

## Abstract

**Background:**

Coronavirus-19 disease is more severe in patients on antiretroviral therapy (ART). Low-income countries, such as those in Sub-Saharan Africa, are particularly vulnerable to the virus’ spread. However, there is little information on antiretroviral therapy (ART) use in Ethiopia during the pandemic, particularly in the study area. Therefore, this study aimed to assess the level of antiretroviral treatment adherence and associated factors during the COVID-19 pandemic era in public hospitals in Jigjiga City, Somalia, and Eastern Ethiopia.

**Methods:**

An institution-based cross-sectional study was conducted among 382 randomly selected HIV patients on antiretroviral therapy (ART) follow-up in public hospitals in Jigjiga City from March 1–30, 2022. The data was collected through face-to-face interviews and a review of the patient’s record. To explore the relationship between variables, both bivariate and multivariable logistic regression analyses were performed. The adjusted odds ratio (AOR) was utilized, along with a 95% confidence interval, to assess the strength and direction of the association. Statistical significance was considered at *p* < 0.05.

**Results:**

The antiretroviral therapy (ART) adherence rate of HIV patients was 76.9% (95% CI, 71.9–82). Disclosing HIV status to sexual partners [AOR = 2.3, (95% CI (1.22–4.19)], having communication with health care providers’ [AOR = 3.2, (95% CI (1.57–6.53)], having no history of current substance use [AOR = 2.6, (95% CI (1.45–4.63)], and patients who did not fear COVID-19 infection [AOR = 5.8 (95% CI (11–10.98)] were significantly associated with antiretroviral therapy (ART) adherence.

**Conclusion:**

In this study, the level of antiretroviral therapy (ART) adherence was poor in comparison to the expected level. Patients’ adherence status was favorably related to disclosing their status to families and having contact with their healthcare providers, whereas worrying about COVID-19 pandemic infection and current substance use was adversely associated.

## Introduction

The human immunodeficiency virus (1) infection continues to be a serious problem for global public health issues (2). Approximately 37.9 million people worldwide are living with HIV, and 27.5 million have access to antiretroviral therapy (3). All of these people are susceptible to COVID-19 infection (4). Low-income countries, including those in sub-Saharan Africa, are most affected by the virus spread (5).

The COVID-19 pandemic has resulted in various barriers to HIV treatment around the world, resulting in AIDS-related mortality and HIV transmission (6). Furthermore, during the COVID-19 outbreak, people living with HIV (PLHIV) had difficulty maintaining HIV treatment adherence and achieving viral suppression, with more than one-third of their medication uptake disrupted (7). There was also evidence that lockdowns during the pandemic led to a decrease in HIV testing and treatment (8).

COVID-19 had a rapid spread and exponential increase in the number of COVID-19 complications in PLHIV, and it has had an impact on healthcare systems around the world by diverting resources from less urgent services to COVID-19 control and management. Consequently, the pandemic had a negative impact on the delivery and maintenance of ART adherence, and the UNAIDS’ 95–95-95 target for 2030 will be challenged (9). Poor ART adherence in high-burden settings could lead to an additional 10% increase in HIV deaths as a result of the COVID-19 epidemic (10). Furthermore, due to the pandemic, 19% of PLHIV did not receive antiretroviral therapy (11). Therefore, PLWH who have difficulty accessing ART and taking their medications on a daily basis may develop drug resistance and elevated HIV viral levels, increasing the risk of viral transmission (12).

On March 13, 2020, the Federal Ministry of Ethiopia confirmed the first case of COVID-19 in Addis Ababa (13). Ethiopia had been preparing to respond to the COVID-19 pandemic since mid-January 2020, and at the end of March 2020, various preventive measures, including physical separation, were implemented, followed by the proclamation of a state of emergency with various community containment measures (14). This was a difficult time for HIV patients as they had to frequently go to the pharmacy for their refills. The Federal Minister of Health has issued guidelines to standardize national responses to COVID-19 control among PLHIV and healthcare workers. Reducing patient contact with health facilities may reduce the burden on these facilities, potentially mitigating the COVID-19 pandemic’s consequences. Models such as 6-Months Multi-Month Dispensing (6MMD) for patients qualified for the appointment spacing model and 3-Months Multi-Month Dispensing (3MMD) for PMTCT, newly identified customers, clients on second-line ART, and unstable clients who do not seek admission (15).

Good adherence to antiviral medication, which is crucial in managing HIV, can be influenced by a range of factors. These factors can be categorized into individual, service-related, and therapy-related aspects. They encompass psychiatric conditions, substance abuse, lack of psychosocial support, social stigma, difficulties in disclosing one’s HIV status, negative effects of drugs, the burden of medication, inadequate healthcare services, and the costs associated with treatment (1, 16, 17). It is important to recognize that these factors can contribute to a decline in adherence, which in turn can lead to the development of drug-resistant HIV. Drug resistance, higher death rates, poor treatment outcomes, the incidence of opportunistic infections, and increased vulnerability to various comorbidities were among the identified factors as obstacles to adherence to ART (18). Therefore, it is essential to identify and address these factors in order to improve adherence (19).

In eastern Ethiopia, there is a scarcity of evidence regarding the effect of the COVID-19 pandemic on HIV treatment adherence and related factors. Therefore, this study aimed to assess the level of antiretroviral treatment adherence and associated factors during the COVID-19 pandemic era in public hospitals in Jigjiga City, Somalia, and Eastern Ethiopia.

## Materials and methods

### Study design, area, and period

A facility-based cross-sectional study was carried out in Jigjiga City, the capital of the Somali region in eastern Ethiopia. It is approximately 650 kilometers from Ethiopia’s capital, Addis Ababa. According to the Ethiopian Central Statistical Agency’s 2007 census, this city had a total population of 763,509 people, including 366,484 men and 397,025 women. The city is one of the most HIV-positive places in the country, and it has seen the majority of COVID-19 cases. Jigjiga City is organized into three woredas, or 30 kebelles: 20 urban and 10 rural. The city was served only by Karamara General Hospital, Jigjiga University Referral Hospital, and Ablele Primary Hospital. The study was carried out from March 1 to March 30, 2022.

### Population, inclusion, and exclusion criteria

The source population consisted of all individuals living with HIV who were receiving antiretroviral therapy, whereas the study population comprised all people living with HIV who were 18 years of age or older and receiving antiretroviral treatment in public hospitals in Jigjiga. Adults aged 18 and older who were taking antiretroviral medicine at the time of data collection and agreed to participate in the study were included.

### Sample size and sampling procedure

The sample size was determined using the single population proportion formula [*n* = (Za/2) pq/d2]. Where n is the sample size, Z is the standard normal deviation set at 1.96 (for a 95% confidence level), d is the margin of a level acceptable (0.04), and p is the rate of ART adherence (78.7%) taken from the study conducted in Western Ethiopia during the COVID-19 pandemic (20). With a 15% non-response rate, the final sample size was 382.

**Figure 1:**
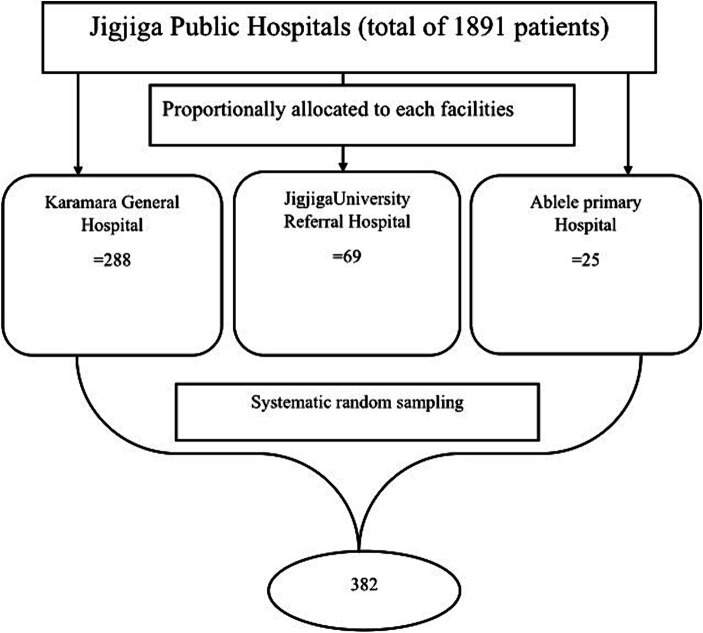
Schematic presentation of sampling procedure to include ART patients attending at public Hospitals, Eastern Ethiopia during COVID 19 pandemic era.

The facility’s monthly ART report indicated that an average of 1891 adult ART users were qualified. To pick samples from each institution, samples were proportionally allotted to each hospital based on their average patient flow for outpatient services over the previous months. Patients that visited the ART clinic at hospitals included 1,425 at Karamara General Hospital, 341 at Jigjiga University Referral Hospital, and 125 at Ablele Primary Hospital. After the sample was proportionally distributed, the systematic random selection approach was used to choose actual participants from each hospital at the fifth interval. All health institutions used a lottery to choose the first person from the interval (Figure 1).

### Data collection methods

A structured questionnaire and patient record review were used to extract information on patient adherence to treatment and the main factors that influence adherence. The questionnaire was developed following a review of numerous and diverse relevant pieces of literature that supported the objectives of this study (20–22). Data were collected using a structured questionnaire, which was prepared first in the English language and translated into local languages (Somali and Amharic) and then back to the English language, which included questions about respondents’ socio-demographic characteristics, COVID-19 containment measures, health system-related factors, and awareness of the COVID-19 pandemic.

Four ART-trained data collectors (2 BSc and 2 diploma nurses) and one BSc health officer supervisor who did not work in the assigned health facilities were recruited. As data collectors, ART-trained interviewers who could speak Amharic and the local language (Somali) fluently were chosen.

Before beginning data collection, the questionnaire was pre-tested at the selected facilities in Wuchale City with 5% of the total sample. Two days before the survey, all data collectors and supervisors were oriented and trained on how to conduct interviews and record data. The data was collected through face-to-face interviews and a review of the patient’s record.

### Operational definitions

#### Adherence

Drug adherence of ≥95% (<2 doses of 30 doses or < 3 doss of 60 doses is missed) (FMOH, 2018).

#### In-adherent

Drug adherence of <95% (3–5 doses of 30 doses or 3–9 doses of 60 doses missed) (FMOH, 2018).

### Data quality assurance

The questionnaire was translated into the local languages. Five percent of the questionnaires were pre-tested 1 week before the actual data collection among adults age 18 and older ART users in Wuchale General Hospital, which is located about 80 kilometers away from the study area. Data collectors were trained for 2 days on the data collection process to have a common understanding. During data collection procedures, all the collected data were reviewed and checked daily for their completeness. The data collectors were closely supervised. Data was entered by two data clerks, and consistency was cross-checked by comparing the two separately entered data sets on EpiData.

### Statistical analysis

The collected data was entered into Epi-data version 3.1 and exported to SPSS software version 26.0 for analysis. The analysis was done using bi-variable and multi-variable logistic regression to observe the effects of independent variables on the outcome variable while simultaneously controlling for other potential confounding factors. Independent variables with a *p*-value <0.25 in the bi-variable analysis were considered for further analysis in the multivariable logistic regression model. Multi-collinearity was checked using the variance inflation factor (VIF). The model’s goodness-of-fit was checked using the Hosmer-Lemeshow test. Adjusted odds ratios with 95% confidence intervals were estimated to show the presence, strength, and direction of associations. Statistical significance was declared at a *p*-value <0.05.

### Ethical considerations

The study was conducted after ethical approval was obtained from the Institutional Health Research Ethics Review Committee (IHRERC), College of Health and Medical Sciences, Haramaya University, with reference number (IHRERC/031/2022). The Somali Regional Health Bureau and the respective hospitals received an official letter of permission from the College of Health and Medical Sciences. Hospital administrators and participants were informed about the nature, purpose, duration, procedure, risks, and benefits of the study. The heads of the hospitals and each study participant then provided informed, voluntary, written, and signed consent before data collection was started. The confidentiality of their responses was maintained throughout the research process. The data were collected in a private setting to prevent the transmission of the COVID-19 pandemic.

## Results

### Socio-demographic characteristics

A total of 373 HIV patients were enrolled in the study, with a response rate of 98%. The median age was 32 (26–39) and 219 (58.7%) were female. Of the total, 230 (61.7%) and 130 (34.9%) were in the age groups of 18–34 and 35–54 years, respectively. Two hundred eleven (55.6%) were married and 99 (26.5%) were single. One hundred eighty-five (49.6%) had attended primary education. One hundred and eighty-three (49.1%) were Muslims, and the median monthly income was 800 (550–1900) with 233 (62.5%) having an average monthly income of less than 1,000 Ethiopian birr (Table 1).

**Table 1:** Sociodemographic characteristics of HIV patients in Jigjiga Public Hospitals March 2022 (*n* = 373).

Variable	Category	Number (%)
Sex	Male	154 (41.3)
	Female	219 (58.7)
Age group (in years)	18–34	230 (61.7)
	35–54	130 (34.9)
	>55	13 (3.5%)
Marital status	Single	99 (26.5%)
	Married	211 (55.6%)
	Divorced	36 (9.7%)
	Widowed	16 (4.3%)
	Separated	11 (2.9%)
Religion	Muslim	183 (49.1%)
	Orthodox	118 (31.6%)
	Protestant	61 (16.4%)
	Catholic	9 (2.4%)
	Other	2 (0.5%)
Level of education	No formal education	33 (8.8%)
	Primary (1–8)	185 (49.6%)
	Secondary (9–12)	127 (34%)
	College and above	28 (7.5%)
Occupation	Student	43 (11.5%)
	Daily laborer	32 (8.2%)
	Merchant	98 (26.3%)
	Government employee	75 (20.1%)
	Private employee	40 (10.7%)
	Farmer	27 (7.2%)
	Unemployed/housewife	58 (15.9%)
Family size	One	64 (17.2%)
	Two	36 (9.7%)
	≥Three	273 (73.1%)
Monthly income	≤1,000	233 (62.5%)
	1,001–3,000	114 (30.5%)
	3,001–10,000	26 (7%)

### Social, behavioral, and clinical characteristics of ART patients

Two hundred ninety-three (78.6%) of the study participants had no family or social support and 330 (88.5%) perceived social stigma. Two hundred eight nine (77.5%) patients disclosed their HIV status to their relatives, 62 (16.6%) had co-morbidity and 53 (14.2%) were currently substance users. Regarding the stage of HIV, 180 (48.3%) were on stage II and 163 (43.7%) were on stage I. Two hundred thirty-five (63%) of the study participants had a treatment duration of 2–5 years and 100 (26.8%) were 6–10 years. The majority of the study participants, 360 (96.5%) had working conditions (Table 2).

**Table 2:** Distribution of study participants by clinical and behavioral variables among HIV/AIDS patients at public hospitals in Jigjiga City, Eastern Ethiopia, 2022 (*n* = 373).

Variable	Category	Frequency
Stage of HIV/AIDS	Stage I	163 (43.7%)
	Stage II	180 (48.3%)
	Stage III	29 (7.8%)
	Stage IV	1 (0.2%)
Family/social support	Yes	80 (21.4%)
	No	293 (78.6%)
Perceived social stigma	Yes	330 (88.5%)
	No	43 (11.5%)
Duration of disease	<1 year	12 (3.2%)
	2-5 year	235 (63%)
	6-10 year	100 (26.8%)
	>10 year	26 (7%)
Current substance use	Yes	53 (14.2%)
	No	320 (85.8%)
Family disclosure status	Yes	289 (77.5%)
	No	84 (22.5%)
Comorbidity	Yes	62 (16.6%)
	No	311 (83.4%)
Patient condition	Working	360 (96.5%)
	Ambulatory	13 (3.5%)

### COVID-19 awareness and effects of containment measures

Of the total study participants, 237 (63.5%) of them had awareness of COVID-19 prevention mechanisms. The proportion of study participants’ responses on maintaining physical distancing to prevent the pandemic was 195 (52.3%). Two hundred seventy (72.4%) said they had communication with their ART care providers during the pandemic period and 162 (43.7%) used Multi-month dispensing methods. Two hundred seventy-four (73.5%) of the study participants lost their work or education, 68 (18.2%) missed feeding schedules due to the COVID-19 pandemic, and 191(51.2%) had fears or worries about COVID-19 infection during their treatment refill. Ninety-four (25.2%) participants got tested and vaccinated for COVID-19, and 289 (77.7%) trusted COVID-19-related information (Table 3).

**Table 3:** COVID-19 awareness and containment measures among participants in public hospitals of Jigjig City eastern Ethiopia, 2022 (*n* = 373).

Variable	Category	Frequency
Awareness of COVID-19 prevention methods	Yes	237 (63.5%)
	No	136 (36.5%)
Used special measures (multi-month dispensing) during the pandemic	Yes	162 (43.4%)
	No	211 (56.6%)
Maintain physical distancing	Yes	195 (52.3%)
	No	178 (47.7%)
Communication with healthcare providers during the COVID-19 pandemic	Yes	270 (72.4%)
	No	103 (27.6%)
Lost work/education due to the COVID-19 pandemic	Yes	274 (73.5%)
	No	99 (26.5%)
Vaccinated for COVID-19	Yes	94 (25.2%)
	No	279 (74.8%)
Missed your feeding schedule due to the pandemic	Yes	68 (18.2%)
	No	305 (81.8%)
Tested for COVID-19 infection	Yes	33 (8.8%)
	No	340 (91.2%)
Trust COVID-19-related information	Yes	289 (77.5%)
	No	84 (22.5%)
Worried/fear of COVID-19 infection during treatment refill	Yes	191 (51.2%)
	No	182 (48.8%)

### Level of adherence to ART therapy

From a total of 382 ART participants, 287 (76.9%) [95% CI: (72.4–80.9)] patients adhered to the prescribed doses of ART medication, and 86 (23.1%) of the participants missed their treatment refill.

### Factors associated with the level of ART adherence

Bi-variable logistic regression was performed to assess the association of each independent variable with the dependent variable adherence status. The result of this study revealed that sex, current substance use, family disclosure of HIV status, communication with the health care provider, and fear of COVID-19 infection were variables that entered multivariable logistic regression.

In multivariable logistic regression, current substance use, family disclosure, fear of COVID-19 infection, and communication with healthcare providers during the pandemic were found to be predictors of the outcome variable. Participants in the study were 3.2 times more likely to adhere if they had no history of current substance use than if they did [AOR = 3.2 (1.57–6.53)]. In comparison to participants who did not disclose, there was a 2.3-fold increase in the likelihood of adherence among those who disclosed their HIV status to their families [AOR = 2.3 (1.22–4.19)]. Participants who had contact with their care providers had 2.6 [AOR = 2.6 (1.45–4.63)] times higher ART adherence than those who did not have communication during the pandemic era. PLWH who did not have fear or worries about COVID-19 infection showed good adherence levels than their counterparts [AOR = 5.8 (3.11–10.98)] (Table 4).

**Table 4:** Bivariate and multivariable logistic regression analysis of missing treatment variable in Jigjiga Public Hospitals, 2022.

Variable	Category	Adherence status		COR (95% CI)	AOR (95% CI)
		Adherent	Nonadherent		
Sex	Male	109	45	0.56 (0.34–0.91)	0.8 (0.46–1.44)
	Female	178	41	1	1
Age	18–34	175	55	0.96 (0.25–3.59)	
	35–54	102	28	1.09 (0.28–4.24)	
	>55	10	3	1	
Marital status	Single	71	28	1	
	Married	168	43	1.54 (0.89–2.67)	
	Divorced	27	9	1.18 (0.49–2.83)	
	Widowed	12	4	1.18 (0.35–3.98)	
	Separated	9	2	1.77 (0.36–8.73)	
Level of education	No formal education	26	7	1	
	Primary (1–8)	135	50	0.73 (0.29–1.78)	
	Secondary (9–12)	102	25	1.1 (0.43–2.82)	
	College and above	24	4	1.6 (0.42–6.22)	
Occupation	Student	33	10	1	
	Government employee	58	17	1.03 (0.42–2.52)	
	Private employee	34	6	1.7 (0.56–5.26)	
	Farmer	19	8	0.72 (0.24–2.14)	
	Merchant	74	24	0.93 (0.40–2.17)	
	Daily laborer	26	9	0.72 (0.07–2.37)	
	Unemployed/Housewife	43	12	1.2 (0.37–5.23)	
Family size	One	48	16	1	
	Two	29	7	1.4 (0.51–3.76)	
	≥ Three	210	63	1.1 (0.59–2.09)	
Monthly income	≤1,000	178	55	1	
	1,001–3,000	90	24	1.2 (0.67–1.99)	
	3,001–10,000	19	7	0.8 (0.33–2.1)	
Residence	Urban	264	77	1.3 (0.59–3.02)	
	Rural	23	9	1	
Family/Social support	Yes	69	15	1.5 (0.81–2.78)	
	No	218	71	1	
Duration of treatment	>1yr	11	1	1	
	1–5yr	181	54	0.3 (0.04–2.41)	
	5–10yr	76	24	0.29(0.04–2.35)	
	>10yr	19	7	0.25(0.03–2.28)	
Current substance use	Yes	26	27	1	1
	No	261	59	4.6 (2.5–8.44)	3.2 (1.57–6.53)
Family disclosure	Yes	233	56	2.3 (1.36–3.94)	2.3 (1.22–4.19)
	No	54	30	1	1
Co-morbidity	Yes	48	14	1.03 (0.54–1.98)	
	No	239	72	1	
Communication with a healthcare provider	Yes	221	49	2.53 (1.52–4.20)	2.6 (1.45–4.64)
	No	66	37	1	1
Fear of COVID-19 infection	Yes	120	71	1	1
	No	167	15	6.59 (3.6–12.06)	5.8 (3.11–10.98)

## Discussion

This study assessed the level of ART adherence and its associated factors among adult PLHIV during the era of the COVID-19 pandemic. The finding of the study was that optimal ART adherence was 76.9% (95% CI, 72.4–80.9), and factors like disclosing HIV status to families and relatives, patients who had contact with their care provider, having no current history of substance use, and not having fear of COVID-19 infection to take ART medication from health faculties had a good association with ART adherence.

According to the findings of the study, the patient’s adherence rate was 76.9%, which is below the WHO goal of good adherence (95%). Local studies conducted during the COVID-19 pandemic close thus study, Addis-Ababa (73.6%) (22) and West Ethiopia (78.7%) (20). The result is above average compared to the systematic review and meta-analysis study done in SSA (72.9%) (23), Nekemt (73.1%) (24), Dire-Dawa (65%) (25), Harar (71.8%) (17) and Benushangul-Gumuz 39.7% (26), but below the study done in Miami, Florida, 83% (27), Tigray, 94.84% (28) and Southwest Ethiopia (83.3%) (21). The variation could be due to the study time since this study was done during the COVID-19 pandemic period and setting.

The study indicated that those who had no history of current substance use (alcohol, chat, cigarette) were 3.2 times more likely to adhere than those who had a history of current substance use. This result agrees with the study done in the Somali Region (29), West Ethiopia, and the Systematic review and meta-analysis study in SSA (30). Substance user patients may be fearful of the community’s norms, culture, and prejudice, as well as the effects of the substance, which can induce behavioral changes and a failure to manage their viral load.

The study revealed more adherence among patients who had contact with their healthcare provider during the pandemic. The study shows those who had communication with healthcare providers were about 2.3 times more likely to be adherent than their counterparts. This is similar to the finding in Addis-Ababa that reported good communication as a predictor of good adherence (22). Having contact with a healthcare practitioner, particularly during the COVID-19 pandemic, may assist patients in reducing their concerns or fear of infection, providing pandemic knowledge, demonstrating standing within any circumstances, and informing them to adhere to the ART.

Patients who were worried about going to health facilities have been less adhered to than those who did not. The study shows those who had no fear of going to a health facility during the COVID-19 pandemic were 5.8 times more adherent than those who had fears or worries. The study was supported by a study conducted in Hawassa (31) and Uganda (3). Furthermore, patients’ worries about visiting medical facilities may be related to their belief that if they become ill, they will be treated unfairly, ignored, and cruelly. Another probable reason for these fears is a lack of personal protective equipment (face masks, sanitizers, and hand washing materials).

There was a statistically significant difference between patients who disclosed their HIV status to their families or relatives and those who did not. The study showed patients who disclosed their status to their families or relatives were 2.3 times more likely to adhere to their treatment compared to those who did not. This finding agrees with the studies by Gondar (32), Harar (17), and East Ethiopia (33) that indicated PLHIV who disclosed their HIV status to their partners or others were 3.6 times more likely to have good adherence to ART. Disclosing HIV status may help patients get direct and indirect support, psychological stability, easy access to medication, and reduce stigma, violence, and discrimination.

### Limitations of the study

Due to the cross-sectional nature of the design, the study does not confirm a conclusive cause-and-effect relationship. The patient’s status was reviewed from their ART registration and follow-up, and treatment continuity was examined; however, due to the lack of availability of some indicators on the registration and assessment, they may not have remembered, which can alter the study’s results. This study also did not evaluate the impact of mental disorders and physical illnesses on ART adherence, despite their known significance. Moreover, the study did not employ diverse methods of data collection that encompass self-reporting method of data collection.

## Conclusion

The level of ART adherence during COVID-19 is below the WHO standard. Disclosing HIV status to families and relatives, patients who had contact with their care provider, had no current history of substance use, and did not fear COVID-19 infection to take ART medication from health facilities were significantly associated with good ART adherence. To improve patients’ capacity to overcome barriers to disclosure and adherence in the course of their treatment, healthcare providers, in particular, and other program supporters at the facility level should have a focus on and strengthen patient-centered adherence counseling and follow and encourage them to disclose their status. Additionally, healthcare professionals or ART clinics would need to improve communication with the PLWH in alarming medication use and appointments, encourage abstaining from substance use (reducing it), and increase the patient’s awareness of COVID-19, which can be done by counseling them at the appointment.

## Data availability statement

The original contributions presented in the study are included in the article/supplementary material, further inquiries can be directed to the corresponding author.

## Ethics statement

The studies involving humans were approved by the Institutional Health Research Ethics Review Committee (IHRERC), College of Health and Medical Sciences, Haramaya University. The studies were conducted in accordance with the local legislation and institutional requirements. Written informed consent for participation in this study was provided by the participants’ legal guardians/next of kin.

## Author contributions

FA: Data curation, Formal analysis, Investigation, Methodology, Software, Validation, Visualization, Writing – review & editing, Supervision. ST: Conceptualization, Formal analysis, Investigation, Methodology, Software, Writing – original draft. BF: Data curation, Formal analysis, Methodology, Software, Supervision, Validation, Visualization, Writing – review & editing. HA: Data curation, Formal analysis, Methodology, Software, Supervision, Validation, Writing – review & editing. BG: Data curation, Formal analysis, Methodology, Software, Supervision, Validation, Writing – review & editing.
